# A systematic review of childhood obesity in the Middle East and North Africa (MENA) region: Health impact and management

**DOI:** 10.12715/apr.2017.4.6

**Published:** 2017-05-15

**Authors:** Nesrine S. Farrag, Lawrence J. Cheskin, Mohamed K. Farag

**Affiliations:** 1 Department of Public Health and Community Medicine, Mansoura University Faculty of Medicine, Mansoura, Egypt; 2 Department of Health, Behavior & Society, and Global Obesity Prevention Center, Johns Hopkins Bloomberg School of Public Health, Baltimore, MD, USA; 3 Epidemiology Department, Johns Hopkins Bloomberg School of Public Health, Baltimore, MD, USA

## Abstract

Childhood obesity has serious consequences both immediately and in adulthood. The rates of obesity in children and adolescents are rising rapidly in the Middle East and North Africa (MENA) region. We systematically searched the literature to explore adverse effects associated with childhood obesity in this region and the management efforts for dealing with it. Inclusion criteria were: English-language, non-basic-science focused articles that used any of the standard obesity definitions and were conducted in the MENA countries within the last five years. We searched PubMed using combinations of key terms ((childhood) OR adolescence) AND obesity) AND (MENA or each country) AND (“last five years” [PDat]). Studies that examined adverse effects of childhood obesity gave fairly consistent results, revealing associations with higher blood pressure, pre-diabetes, metabolic abnormalities, and cardiovascular risk. Little or no overall effect on rates of childhood obesity has yet been demonstrated by interventions used to manage the problem. Obesity has a considerable impact on the health of children and adolescents, and the countries of the MENA region should endorse strategies and programs to prevent and manage this problem in an effective way.

## Introduction

Childhood obesity, being associated with a higher risk of developing many non-communicable diseases (NCDs) at a younger age as well as with premature death in adulthood, is a serious public health problem and one of the most important public health challenges of the 21st century. The risk of NCDs resulting from obesity depends in part on the age of onset and duration of obesity (Kelsey, et al., 2014; Litwin, 2014). Worldwide, the number of overweight children under the age of five was estimated to be over 42 million in 2013. Most (about 31 million) of these children live in developing countries [1].

The World Health Organization (WHO) reported that the growing level of obesity among children and adolescents in all Eastern Mediterranean countries should be taken very seriously, especially with evidence that a nutrition transition has occurred in this part of the world. Nutrition transition refers to the increased consumption of unhealthy foods in conjunction with an increased prevalence of overweight in low- to middle-income countries. Nutrition transition occurs side-by-side with epidemiological transitions including urbanization, the shift to a modern lifestyle, shifts in the quality of the diet with special reference to fast food, and expansion in the use of modern transportation and modern technologies leading to a more sedentary lifestyle. Nutrition transition is associated with increasing obesity rates all over the world, and especially in middle income countries, and has serious consequences in terms of public health outcomes, economic growth, and international nutrition policy [2].

**Figure 1:**
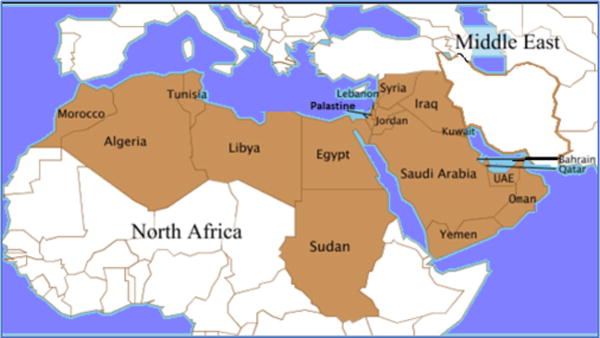
Map of the Middle East and North Africa (MENA) countries

This review included 18 countries which constitute the vast majority of the Middle East and North Africa (MENA) region: Algeria, Bahrain, Egypt, Iraq, Jordan, Kuwait, Lebanon, Libya, Morocco, Palestine, Qatar, the Kingdom of Saudi Arabia (KSA), Sudan, Syria, Tunisia, the United Arab Emirates (UAE), and Yemen (Fig. 1).

The objectives of this review were to examine the adverse effects associated with obesity in the MENA region and to examine the efforts made thus far to manage the problem. This review complements our recent comprehensive systematic review of the latest studies reporting the prevalence of overweight and obesity among male and female children and adolescents under the age of 20 in the countries of the MENA region, and its underlying risk factors.

## Methods

**Figure 2:**
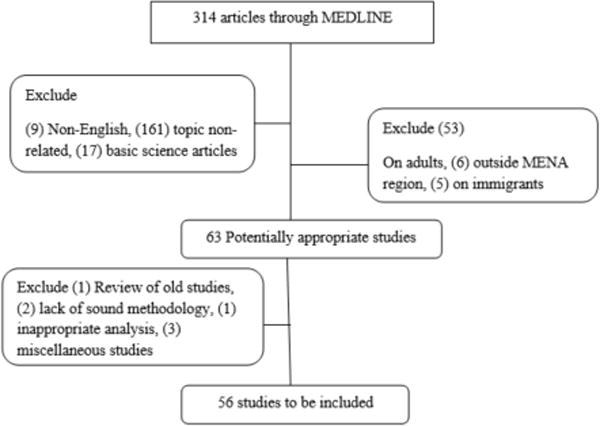
Flow chart of the screening process used to gather articles included in the review

Data were gathered from original research articles and systematic review articles of overweight and obesity in childhood and adolescence in the countries of the MENA region as a primary focus, or as a risk factor for other problems (Fig. 2).

We set the following inclusion criteria: articles written in English; non-basic science focused; used any of the commonly-used standards in the diagnosis of overweight and obesity: the International Obesity Task Force (IOTF), World Health Organization (WHO), and Centers for Disease Control and Prevention (CDC) standards; and were conducted in the MENA countries within the last five years, whether reviews, observational, or interventional studies. We searched PubMed using combinations of key terms ((childhood OR adolescence) AND obesity) AND (MENA or each country) AND (“last five years” [PDat]).

The search strategy yielded 314 papers. The details of the screening process of articles are in our companion article (*Advances in Pediatric Research*, – in publication). Fifty-six articles remained and were included in our review. They covered Algeria [3], Bahrain [1], Egypt [2], Jordan [4], Kuwait [4], Lebanon [5], Morocco [1], Palestine [1], Qatar [3], Saudi Arabia [14], Sudan [1], Syria [1], Tunisia [2], UAE [5], Yemen [2], and multi-country studies [3]. All studies were cross sectional, except for 3 reviews, 2 RCT, 1 quasi-experimental study and 1 retrospective cohort study.

## Results

### Obesity-associated adverse effects

#### Cardiovascular risk

Rizk and Yousef reported that Qatari children who were overweight and obese (n=315) were at increased risk of cardiovascular disease in adulthood [3]. They found that children’s overweight and obesity significantly increased the odds ratios of cardiovascular risk factors, namely hypertriglyceridemia (OR 6.34, CI 2.49–13.44, p<0.0001), LDL-C (OR 3.18, CI 1.04–9.75, p=0.043), hypercholesterolemia (OR 1.88, CI 1.10–3.19, p=0.020), and increased waist circumference (OR 1.40, CI 1.29–1.55, p=0.022). Overweight and obese Qatari children were at significantly higher risk of atherosclerosis (assessed by atherogenicity index) by about two-fold (OR 1.83, 95% CI 1.06–3.15, p=0.025) [3].

#### Dental decay

Goodson et al. found an inverse relationship between obesity and dental decay in Kuwaiti children (mean age 11.36 years) [6]. This finding contradicts the obesity-sugar and the obesity-dental decay relationship hypotheses for reasons that are not currently clear. The study showed a statistically significant dose-response pattern between dental decay and BMI: the percentage of decayed or filled teeth decreased from 15.61% (n=193) in underweight children, to 13.03% (n=4,094) in normal healthy weight children, to 9.73% (n=1,786) in overweight children to 7.87% (n=2,202) in obese children. Although boys had more dental decay than girls, the reduction of tooth decay as a function of BMI was greater in boys [6].

#### Diabetes

Salameh and Barbour found a significant increase in the risk of pre-diabetes (relative risk=4.93) and diabetes (relative risk=2.85) in overweight versus normal weight Lebanese adolescents aged 11–18 (p=0.001) [5]. Mamtani et al.’s study of Qatari adolescents aged 11–18 (n=**1694**) also found that on bivariate analysis, waist-to-height>0.5 and obesity (WHO 2007 reference) were significantly associated with pre-diabetes (p=0.01 and 0.05, respectively), but in a multivariate model, having a waist-to-height ratio>0.5 (OR 1.8, 95% CI 1.1–3.0) was the only variable significantly associated with the risk of pre-diabetes [4].

#### Disordered eating attitudes

Disordered eating was defined by the American Psychiatric Association’s Diagnostic and Statistical Manual of Mental Disorders (DSM-IV) as “severe disturbances in eating behavior.” This definition was further widened by Fairburn and Walsh who defined it as “a persistent disturbance of eating behavior or behavior intended to control weight, which significantly impairs health or psychosocial functioning. This disturbance should not be secondary to any recognized general medical disorder or any other psychiatric disorder” [7]. A cross-sectional study was conducted in seven cities in Arab countries, namely Algeria, Jordan, Kuwait, Libya, Palestine, Syria, and the UAE, as a part of the ARAB-EAT Project. The study explored the association between obesity and disordered eating attitudes (EA) among adolescents aged 15–18 years. The Arabic version of the Eating Attitudes Test (EAT-26) was validated by Al Subaie et al. who reported a good internal consistency (Carmine’s theta 79.6%) [8]. The study found a high prevalence of disordered eating attitudes (EA) in the participating Arab countries, ranging from 13.8% to 47.3% among males, and 16.2% to 42.7% among females, with a significant difference between genders (p<0.0001). The risk of disordered EA among adolescents who have obesity was two to three times higher than that of the non-obese of both genders in all countries except for Algerian males, Kuwaiti females, and Palestinian males. Apart from Algerian males and Palestinian adolescents, these associations were statistically significant. The highest association with obesity in both genders was evident in Libyan adolescents, where ORs (95% CIs) were 3.54 (1.81–6.91), and 3.07 (1.88–5.03) for males and females, respectively [9].

#### Health-related quality of life

Boodai and Reilly investigated the impact of obesity on the health-related quality of life (HRQL) of 500 Kuwaiti adolescents aged 10–14 years [10]. They found that obesity was not significantly associated with HRQL in regression analysis in a pair-matched comparison of 98 pairs of obese adolescents versus normal-weight peers. The participants were pair matched for sex, school, school year, and ethnic group. Impairment of HRQL was significant only for the physical functioning score (95% CI = −1.5, −9.4), not for psychosocial score or total score. Boodai and Reilly speculated that cultural differences in attitudes towards obesity may explain the lack of significant association between obesity and HRQL among Kuwaiti teens, in contrast to attitudes commonly found in studies conducted in Western societies [10]. On the other hand, Al-Akour et al. found that after adjusting for other variables in a multivariate analysis, overweight and obese Jordanian adolescents (n =1433) in Irbid City had significantly lower average scores on the psychosocial health summary scale and its dimensions, as well as the physical functioning scale (p<0.001) [11].

#### Hypertension

Consistent results have been reported regarding the association between obesity and hypertension in the MENA region. Salman et al. found that, after adjustment for gender and family history, Sudanese children who were obese had a very high relative risk of systolic hypertension (14.7) compared to their normal-weight peers (p<0.01) [12]. Similarly, Aounallah-Skhiri et al. found a significant association between obesity and hypertension in Tunisian adolescents: obesity versus no excess weight markedly increased the prevalence of frank hypertension (boys OR=8.4 [3.7–18.8], girls OR=7.2 [3.2–16.1]), as well as the risk of elevated BP (boys OR=3.7 [2.0–6.9], girls OR=3.4 [2.1–5.6]). Similar though weaker associations were observed with overweight [13].

Badi et al. found that BMI was significantly associated with systolic blood pressure (SBP) and diastolic blood pressure (DBP) in Yemeni children [14]. Age and BMI explained about one-third of the variation in the model for SBP (R2=0.29), and about one-sixth of the variation in the model for DBP (R2=0.16), with the child’s BMI making a higher contribution than age for both SBP and DBP [14]. Mahfouz et al. found a lower risk, albeit still significant, among adolescents in the Aseer region of Saudi Arabia [14]. Using logistic regression analysis, they found that male gender (aOR=2.992, 95% CI=1.933–4.742) and obesity (aOR=2.995, 95% CI =2.342–3.991) were significant risk factors in developing high blood pressure among adolescents [15]. Finally, Abdulle et al. found that high BP was strongly related to body weight among Emirati children and adolescents aged 6–17 years in Abu Dhabi, and that BP appeared to be more strongly associated with BMI (SBP, r=0.34, p<0.001; DBP, r=0.21, p<0.001) than with waist circumference (SBP, r=0.255, p<0.01; DBP, r=0.175, p=<0.01) [16].

#### Injury severity

While some studies outside the MENA region have reported otherwise, a retrospective cohort study of 933 children aged 2–18 years admitted to the King Abdul Aziz Medical City in KSA between May 2001 and May 2009, found that limb and thoracic spine injuries were significantly higher in children who were obese and overweight versus lean children (p=0.041 and 0.012, respectively). But after controlling for age, obesity was not a predictor of injury severity score (lean versus obese, OR −0.35 [−0.9104–0.2102], p=0.2207) and after controlling for age and injury severity score, obesity was not a significant predictor of death as a result of trauma (overweight and obese versus lean, OR 0.85 [0.09–8.17], p=0.88) [17].

#### Metabolic abnormalities

Metabolic syndrome is defined as the presence of at least three components: High blood pressure (SBP or/and DBP≥sex-, age-, and height-specific 90th percentile), abdominal obesity (waist circumference, WC≥sex- and age-specific 90th percentile), hypertriglyceridemia (TG≥110 mg/dL), high fasting glucose (FBG≥110 mg/dL), and low high-density lipoprotein-cholesterol (HDL-C<40 mg/dL) [18]. According to Khader et al., children and teens aged 7–18 years with obesity (n=665) had a significantly higher prevalence of metabolic syndrome (MetS) compared to non-obese subjects (13.3% versus 3.6%, p<0.001) [19]. Factor analysis was performed on BMI, WC, SBP, DBP, HDL-C, TG, and FBG to produce the minimum number of variables that accounted for most of the total variance in the data. This study found that obesity was responsible for the largest proportion of the total variance in the clustering of metabolic syndrome (28.4% and 35.5% of the variance in male children and adolescents, 37.1% and 30.6% of the variance in female children and adolescents, respectively). Obesity thus appeared to be a powerful correlate of cardiovascular risk in children and adolescents [19]. In a subsequent study, Khader et al. reported that after adjusting for gender and age, obesity in children aged 7–18 was significantly associated with increased odds of having high triglycerides (OR 3.4 [2.11–5.5]), low HDL-cholesterol (OR 2.3 [1.5–3.7]), and at least one metabolic abnormality (OR7.8 [3.8–15.7] [20]. In Al Ain, UAE, Mehairi et al. found that the prevalence of MetS among 12–18 year old adolescents increased with BMI and was as high as 59% among boys who had obesity [21].

After multivariable adjustment, boys who were overweight (aOR 2.72 [1.37, 5.35]), or obese (aOR 12.70 [7.31, 22.05]), were significantly more likely to have MetS. Similarly, girls who were overweight (aOR 4.23 [1.32, 13.62]) or obese (aOR8.32 [2.73, 25.32]) were also more likely to have MetS [21]. Benmohammed et al. found that among obese Algerian adolescents, prevalence of MetS increased significantly across BMI classes for all definitions of MetS [22]. At 13%, the prevalence of MetS was highest among obese adolescents according to the Cook [23] and De Ferranti [24] definitions.

#### Psychological problems

Salameh and Barbour found that among Lebanese adolescents, obese individuals had significantly higher mean psychological distress scores than individuals at-risk of obesity, and boys were significantly less likely than girls to be distressed by their obesity (p=0.005) [5]. Abdelalim et al. found that there is no significant association between obesity and academic performance in the classroom setting among boys in Kuwait after adjusting for cofounders (mother’s educational level, father’s educational level, and nationality) [25].

Studying the relationship between obesity and body image, Musaiger et al. found that a high proportion of Emirati adolescents (12–17 years) who had overweight and obesity considered themselves average weight (45.0% and 56.9% for males, 52.3% and 46.4% for females, respectively) [26]. The study found that a quarter of overweight males and nearly a third of overweight females reported being forced by their parents to lose weight. Being forced to lose weight was reported at a still higher rate by obese males (52.8%) and females (53.6%) (p<0.001). Also, more than a third of overweight and about half of obese adolescents reported being teased about their weight status by friends (p<0.001). A significantly higher proportion of overweight and obese adolescents had undertaken a weight-loss diet (51.7% and 63.9% in males and 70.5% and 68.1% in females, respectively) [26].

### Nutrition transition

Zaghloul et al. used a single 24-hour recall assessment of dietary intake among children and adolescents in Kuwait [27]. A large proportion of Kuwaiti children showed overconsumption of energy that ranged from 31.5% in males 14–18 years old to 72.2 % in females 4–8 years old. Overconsumption of carbohydrates affected more than 95% of children in all age groups. With the exception of children aged 1–3 years, almost a third of the sample exceeded the upper limit of the acceptable macronutrient distribution range (AMDR) for fat (35% of total energy) [28], and there was marked under-consumption of n-3 and n-6 fatty acids across all age and gender groups. Mean fiber intake was also less than the recommended value for all age groups in both genders [27].

Ng et al. studied the nutrition transition in the UAE and documented that a large percentage of Emirati children (43.2% of girls and 38.2% of boys aged 6–10 years) consumed more calories than needed [29]. About a third of Emirati adolescent females and 16% of adolescent males also consumed more calories than needed. About one quarter of all calories were consumed between meals by Emirati children. Calories from beverages appeared to be one of the major contributors to total calories, comprising up to 14% for boys. Three quarters of female Emirati adolescents spent>5 hours sitting per weekday, compared to less than 50% of male Emirati adolescents [29].

### Management of childhood obesity in the MENA region

Boodai et al.’s study was the first obesity treatment trial in Kuwait [30]. This randomized controlled trial (RCT) tested whether a good practice intervention for the treatment of adolescent obesity would have a greater impact on weight status, waist circumference, and blood pressure than a referral to primary care (control group) for adolescents in Kuwait City. The intervention aimed at reducing sedentary behaviors, increasing physical activity, and improving diet. The study initially included 82 obese children aged 10–14 years that were randomized to either the intervention group or a control group.

The control group was advised to seek help from their primary care provider. The adolescents in the intervention group received 6 sessions of group discussion led by a physician with specialist training in nutrition and a dietician. They attended the sessions with one parent. The intervention lasted for 24 weeks and each session was one hour duration. The study showed that treatment had no significant effect on BMI Z-score relative to control, and resulted in no significant changes in waist circumference and blood pressure. The study highlighted the need to engage obese adolescents and their families in the interventions being studied, and the need for longer term obesity treatment trials [30].

Due to its high prevalence of obesity and consequent diabetes, Kuwait has undertaken a partnership between their health care system, educational resources, industry, and government called the Kuwait–Scotland eHealth Innovation Network (KSeHIN). The aim of this network is to deliver a package of clinical services, education, and research supported by a comprehensive informatics system, which includes a disease registry for children and adults with diabetes. The national childhood registry (CODeR) enrolls about 300 children a year [31].

In Lebanon, Habib-Mourad et al. conducted a pilot study to evaluate the feasibility and effectiveness of the Health-E-PALS intervention [32]. The Health-E-PALS intervention is a recent multi-component, culturally-sensitive intervention to promote healthy eating and physical activity based on social-cognitive theory. Health-E-PALS was delivered to school children aged 9–11 years in the form of 12 weekly classroom sessions, a family program, and a food service intervention targeting school cafeterias and food shops, as well as children’s lunchboxes provided by the family. Students in the intervention group reported purchasing and consuming fewer chips and sugar-sweetened beverages post-intervention compared with controls (p<0.001), and knowledge and self-efficacy scores increased for the intervention (+2.8 and +1.7 points respectively, both p<0.001), but not for the control group. The intervention had no effect on physical activity and screen time, and no changes were reported in BMI between groups post-intervention [32].

A three-year school-based intervention study was conducted in Sousse, Tunisia [33]. All middle schools in the regions of Jawhara and Riadh were assigned to the intervention group while all middle schools in the delegation of Msaken were assigned to the control group. The intervention program consisted of educational sessions as well as lifestyle changes. Some children were selected to be leaders and were trained to participate in implementation of the program, while teachers were trained to lead educational sessions. Students were also encouraged to participate in physical activity through organized after-school soccer games within and between schools. Healthy eating habits were encouraged through offering healthy food alternatives in school snack stores, and children who chose healthy snacks were rewarded with incentive stickers which they collected for a price at the end of every month.

Evaluation of the program was in the form of pre- and post-intervention questionnaires that were administered to 4,003 schoolchildren. The post-intervention questionnaire showed that fruit and vegetable intake significantly increased in the intervention group from 30.0% to 33.2% (p=0.03) while there was a significant decrease in fruit and vegetable intake in the control group from 40.2% to 35.0% (p=0.001). There were also significant BMI shifts within the intervention group, where the number of students in the normal weight category (p=0.03) increased with a concomitant decrease of the number of students in the overweight category (p=0.03) [33].

## Conclusions

Studies that examined adverse effects associated with childhood obesity in the MENA region yielded fairly consistent results. They showed that childhood obesity is associated with higher blood pressure, pre-diabetes, metabolic abnormalities, and cardiovascular risk. Studies also confirmed that obesity was associated with psychological stress, but its effect on HRQL was variable. The studies addressing dental decay and injury scores showed negative results. Dental decay was found to have a significant inverse relationship with BMI; also BMI was not a significant predictor of the injury severity score or death due to trauma.

While approaches to preventing and treating childhood obesity in the MENA region are steadily becoming more widespread and varied, there has been little or no overall effect shown or demonstrated on rates of childhood obesity demonstrated. In fairness, we would point out that the same can be said of interventions in the developed world. Existing programs that have been described in the literature include approaches such as group sessions to reduce sedentary behavior, increase physical activity, and improve diet; a family program; and a food-service intervention targeting school cafeterias and food shops. Such programs seem to work best when they intervene across multiple levels, which include changes at the individual- and family level as well as changes in policies and government spending. Although a treatment trial in Kuwait gave negative results, application of the Health-E-PALS intervention in Lebanon was promising as it showed at least intermediate favorable outcomes, such as knowledge and engagement in change processes. The school-based intervention in Sousse, Tunisia, also showed promising results in terms of changing eating habits and preventing excess weight among adolescents [33].

Habib-Mourad and Ghandour made several evidence-based recommendations based on their pilot study applying the Health-E-PALS intervention in Lebanon [34]. Their recommendations included: (1) making educational material informative, but also fun, interactive, and relevant to the population; (2) better integration with the school curriculum for improved observation and re-assessment, and perhaps, more positive behavioral changes; and (3) collaboration and involvement of various stakeholders is essential for streamlining the implementation of a program and ensuring its success [34]. Also, Musaiger et al. suggested that the school health education curriculum should include information related to healthy body weight and appropriate diet and lifestyle so as to minimize the risk of developing a distorted body image among adolescents [26].

There is sufficient evidence of efforts in the MENA region to aggressively address the problem of childhood obesity. Although the problem is far from being under control, there is an encouraging engagement by a number of countries at various levels of society. The Kuwait–Scotland eHealth Innovation Network (KSeHIN) is one good example of this commitment.

It is important to persist in efforts to reduce the prevalence of childhood obesity. Continued tracking of the magnitude of the problem of childhood obesity, its consequences and potential solutions, and dissemination of evidence-based information to decision makers at government, education, industry, and other levels of influence will help achieve this goal.
